# A Qualitative Investigation of Adolescents’ Perceived Mechanisms of Change from a Universal School-Based Depression Prevention Program

**DOI:** 10.3390/ijerph110505541

**Published:** 2014-05-22

**Authors:** Ian Shochet, Roslyn Montague, Coral Smith, Mark Dadds

**Affiliations:** 1 School of Psychology and Counselling, Queensland University of Technology, Brisbane 4059, Australia; E-Mail: coral.smith@qut.edu.au; 2 New South Wales Institute of Psychiatry, Sydney 2145, Australia; E-Mail: ros.montague@nswiop.nsw.edu.au; 3 School of Psychology, University of New South Wales, Sydney 2052, Australia; E-Mail: m.dadds@unsw.edu.au

**Keywords:** prevention, universal interventions, adolescence, depression, school-based interventions, resilience

## Abstract

A recent meta-analysis provides evidence supporting the universal application of school-based prevention programs for adolescent depression. The mechanisms underlying such successful interventions, however, are largely unknown. We report on a qualitative analysis of 109 Grade 9 students’ beliefs about what they gained from an evidence-based depression prevention intervention, the Resourceful Adolescent Program (RAP-A). Fifty-four percent of interviewees articulated at least one specific example of program benefit. A thematic analysis of responses revealed two major themes, improved interpersonal relationships and improved self-regulation, both stronger than originally assumed. A more minor theme also emerged—more helpful cognitions. It is postulated that both improved interpersonal relationships and improved self-regulation are likely to enhance one another, and more helpful cognitions may express its contribution through enhanced self-regulation. These findings broaden our understanding of the impact of depression prevention programs, beginning to illuminate how such programs benefit participants.

## 1. Introduction

Depression prevention interventions are particularly appropriate for use with adolescents, with depression considered one of the most common mental health problems of this developmental phase [1,2,3]. Many programs exist that target the socio-emotional health of adolescents in order to prevent depressive disorders and a recent meta-analysis supports the universal application of these programs [4]. Despite this evidence, the mechanisms that underlie successful prevention interventions with adolescents are still largely unclear. The question remains whether we accurately understand the impact of such programs. In this study we examined the impact of an evidence-based depression prevention intervention, the Resourceful Adolescent Program (RAP-A), from a qualitative perspective. This methodology allowed the exploration of perceived mechanisms of change that may underlie the program’s impact, as well as a wider range of outcome variables than quantitative methods allow.

There are both benefits and difficulties associated with applying interventions in a universal or targeted manner. One key advantage of universal approaches is the population health benefits; many people exposed to a low risk (e.g., sub-clinical depressive symptoms) can ultimately generate more clinical cases than a group of fewer individuals exposed to a higher risk [5]. Further, the increased recruitment and reach of the universal approach can bolster the impact of such interventions. Abrams and colleagues [6] argue that the impact of prevention is best judged on the product of the effect and the recruitment rate or reach, and not simply the effect size. There are also benefits in preventing even sub-clinical symptoms of depression in previously healthy teenagers, as these symptoms correlate significantly with a range of adverse outcomes and tend to persist for many years [7]. Universal interventions also alleviate concerns regarding stigma. In selective or indicated programs adolescents are singled out for intervention, and screening that labels individuals as “at risk” may lead to stigmatization e.g., [8]. Finally, the opportunity for symptomatic teenagers to experience positive peer modelling is another reason to take a universal approach to intervention [9]. 

Despite these positives, universal programs do have their disadvantages. It has been suggested that some universal programs may be unappealing to the public and politicians, and may be unnecessarily expensive [10]. Other potential disadvantages include a relatively small benefit to the individual, difficulty in detecting an overall effect, and the risk of a perception amongst low-risk individuals that the intervention is of little benefit to them [10]. 

Compared to universal interventions, targeted programs have the advantage of potentially addressing problems earlier, and it has also been argued that it may be most efficient to direct available resources towards high risk groups [10]. However, targeted interventions include the possibility of labelling and stigma, as discussed as an advantage of universal programs. Furthermore, the screening required to identify at-risk individuals can be costly and difficult and must be conducted continually to detect high risk individuals as they emerge [11]. Other screening difficulties include boundary problems, instability of risk status, and difficulty in accurate targeting [10]. Finally, targeted intervention may also lead to ignoring the social context as a focus of intervention. Overall, it seems likely that a mix of universal and targeted intervention may be optimal [10]. 

The most recent meta-analysis exploring the effectiveness of depression prevention interventions in children and adolescents reported positive findings regarding universal programs [4]. Specifically, universal interventions were shown to reduce clinically significant depressive episodes and depressive symptoms both post-intervention and at a three to six month follow up, compared to no intervention. This Cochrane review finding is more positive than those of earlier meta-analyses that reported mixed findings in relation to universal programs e.g., [12,13]. Merry and colleagues [4] emphasize that it has been more difficult to show effectiveness in universal studies than in targeted programs. As such, their collation of evidence supporting universal interventions is a very notable finding in the field.

Despite the evidence supporting prevention interventions, it remains unclear whether program outcomes are attributable to assumed mechanisms of change. Collins and Dozois [14] reviewed depression prevention programs and concluded that the mediators of intervention outcomes have not yet been empirically established. They suggest that it is necessary to isolate mechanisms of change so as to develop more potent prevention programs. Such clarity would also contribute to greater efficiency in the dissemination of active program components [15]. 

A few studies have attempted to investigate mechanisms of change in depression prevention program for adolescents, although with mixed results. Horowitz and colleagues [12] compared the efficacy of two adolescent depression prevention programs—a cognitive behavioral program (CB) and an interpersonal psychotherapy adolescent skills training program (IPT-AST)—and a control condition. Of particular interest is their inclusion of mediation hypotheses related to attribution style, coping skills, perceived quality of the parent-adolescent relationship, and cognitive-behavioral knowledge. Although statistically non-significant, attribution style trended in the expected direction with participants in the CB group reporting a less negative attribution style than those in the control group. No significant effects were found for coping skills between the groups. With regards to cognitive behavioral knowledge, the CB group performed better than the IPT-AST and control groups. No significant differences were found in relation to perceived conflict with mother or fathers. 

Pössel and colleagues [16] evaluated the effects of a cognitive behavioral program (CB) compared to two control conditions – a non-specific control that was structurally equivalent to the CB condition (NSp), and a non-intervention control condition (NIC)—in order to explore whether observed effects are due to specific intervention components or non-specific change mechanisms. At four months follow-up, those in the CB condition had significantly lower scores on a measure of depressive symptoms than both control groups, suggesting that specific factors, in addition to non-specific factors, play a role in intervention outcomes. Future studies that measure potential mediator variables are necessary and the qualitative methodology used in the current study may assist in identifying potential mediator variables to explore further.

The program investigated in this study is RAP-A, a resilience program that has been researched systematically since its development in 1997 e.g., [17,18,19] and endorsed by the Commonwealth Government as a program for preventing adolescent depression [9]. RAP-A is innovative as it integrates elements of both interpersonal approaches and Cognitive Behavior Therapy (CBT). This combination ensures that RAP-A addresses a wide range of psychosocial risk and protective factors for depression, at the individual level as well as interpersonally. CBT and interpersonal psychotherapy are two of the best-supported psychosocial interventions for adolescent depression [20], with research demonstrating both to be better than wait-list or treatment-as-usual approaches [21]. 

We report on a qualitative analysis of what participants believe they gained from a universal depression prevention program, RAP-A. We examined the lived experience of program participants by conducting brief individual interviews. We propose that there is value in extending our understanding of universal interventions and that exploring an integrative program such as RAP-A will broaden our knowledge of the underpinnings of efficacious and effective universal programs. We also propose that there is more to be gained from universal interventions than the quantitative data has shown us thus far. 

## 2. Method

### 2.1. Participants

Participants in the current study were a sample of Grade 9 students who received the intervention condition in a larger study. The larger study included three intervention schools where students participated in RAP-A as part of the school curriculum, and three comparison schools where students engaged in lessons as usual (N = 1,003). Of the 522 students who received the intervention in this larger study, 109 students were selected to participate in the current qualitative study. These participants were selected by a year level coordinator at each school, who was asked to randomly select approximately one-fifth of the students who participated in the RAP intervention. It is noted that this method of participant selection was unlikely to be truly random; but was selected to ensure ease of recruitment for the involved schools. Participants attended one of three Catholic secondary schools (one co-educational, one all-girls, and one all-boys) in Western Sydney, Australia. Sixty males and 49 females were included, with an average age 13.98 years (*SD* = 0.40 years). More males were included in the study as a result of different distributions of participant numbers at the single sex schools. 

### 2.2. Procedure and Materials

This research received the appropriate ethical clearance from the University Human Research Ethics Committee and complies with the Australian National Health and Medical Research Council ethical standards.

#### 2.2.1. Resourceful Adolescent Program (RAP-A)

All participants in the current study participated in RAP-A, an eleven-session universal program for 12 to 15 year olds that promotes resilience and positive coping for the prevention of depression. It is designed to be implemented as part of the school curriculum in classroom groups of approximately 15 students. In the current study each group included 12 to 15 students. The content and process of each session of RAP-A is specified in a Group Leader's Manual [22] and participant workbooks [23] are provided to each student in the program. Cognitive-behavioral techniques include keeping calm, cognitive restructuring, and problem solving. Interpersonal components included in the program emphasize promoting harmony, dealing with conflict, and developing an understanding of the perspectives of others. Techniques that enable the adolescent to maintain self-esteem in stressful situations are also an integral part of the program.

The schools participating in the larger study requested that the program be delivered over five double periods (*i.e.*, two sessions per group meeting) as they were unable to guarantee ten separate meeting times at weekly intervals within one school term. Leaders of each RAP-A group were either health professionals (e.g., psychologists, social workers, nurses; 56%) from local mental health facilities or teachers who had volunteered to participate (44%). An accredited trainer conducted six-hour training of all leaders and supervision and debriefing sessions were routinely held. 

#### 2.2.2. Interviews

Short structured interviews (5 to 10 min duration) were conducted with each participant three months after completion of RAP-A. The interviewer was not previously known to the students, thus reducing the likelihood of students' need to please the interviewer. The taped interviews were transcribed. Responses to the following questions were analysed in this study: “Can you give me specific examples of when you have used skills from the RAP program?” and “Have other people noticed any changes in you?” These questions were selected as they elicited the perceived changes resulting from program participation. The interviewer also asked briefly about what the participants liked most about the program, what they didn’t like and would change, and what they remember RAP-A being about.

#### 2.2.3. Analysis of Data

The transcribed interviews were analysed using thematic analysis, see [24]. This method was deemed appropriate for summarising the data and identifying patterns in the data in order to provide an interpretation. The six steps outlined by Braun and Clarke [24] were followed. Authors familiarized themselves with the data to gain a solid grasp of the interview content. This was done by thoroughly reading, rereading, and annotating the material with preliminary ideas. Initial themes were generated based on inferences from the data, and it was ensured that they were also meaningful in relation to each other. The data was then categorized into these themes. Two themes were considered too broad given the large number of responses they encompassed. Accordingly, sub-themes were developed in order to more clearly capture the data. The themes and sub-themes were reviewed and refined at the level of the coded data extracts as well in relation to the broader data set. Overall themes and sub-themes were then defined and named. A second individual separately categorized the data to ensure agreement. A small number of differences were found and were resolved through discussion. The connections between themes and sub-themes were considered and summarized in a thematic map. Finally, the report was produced.

## 3. Results

Students were able to identify a range of changes they have experienced following participation in RAP-A, with 54% of participants able to nominate at least one specific change they have encountered as a result of their participation. Slightly more girls (61%) than boys (47%) were able to nominate a change. A further 8% stated that the program was beneficial but were unable to nominate specific examples. More boys (13%) than girls (2%) fell into this category. Approximately 25% of participants were either unsure whether they had experienced any changes following their participation in RAP-A or stated that they had not used the skills much or at all. The remainder comprised 7% who stated they had not encountered a situation requiring the use of RAP-A skills and 3% who claimed to possess relevant skills prior to RAP-A participation. 

**Figure 1:**
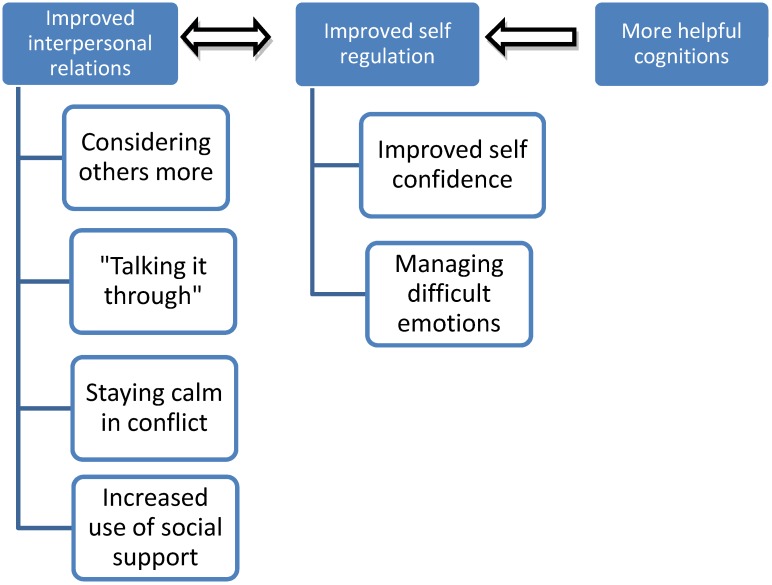
Themes and sub-themes of perceived changes attributed to participation in RAP-A.

Identified changes that were attributed to program participation were analysed further and themes extracted from the data are summarized in Figure 1. As can be seen, two strong themes emerged: improved interpersonal relations and improved self-regulation; and one less robust theme—more helpful cognitions. These first two themes are further broken down into subthemes. Table 1 displays the number of interviews with adolescents during which each theme and sub-theme was identified.

### 3.1. Improved Interpersonal Relations

A very commonly identified theme was improved relationships with peers and/or family members. This theme captures changes such as an increase in empathy, the acquisition of skills promoting calm and effective discussions, and greater utilisation of social support. Given the wide variety of responses comprising this broad theme, sub-themes were developed.

#### 3.1.1. Improved Empathy

Many participants reported that they now have greater consideration for others’ feelings, experiences, and perspectives. For example, “*I think about how others feel more—how they’re feeling*” and “*If someone says something, you just think of what place they’re in and you’re thinking*”, “*That’s their opinion, so you take that into consideration and respect that*”. These responses suggest an improved ability to empathize with others and in the case of the latter response, to use this to modify their own response to another person or situation. Responses highlighting the use of empathy in relation to potential disagreements were also given by a number of respondents, for example, “*I just don’t think, “Oh yeah, I feel bad”, but how (is) that other person feeling?*” and “*(I think)*”, “*Okay, what are they going through?”...before it used to always be my way*”.

**Table 1:** Number of interviews with adolescents during which themes were identified as specific outcomes of RAP-A participation.

Theme	Female		Males		Total	
	n	%	n	%	n	%
**Improved interpersonal relationships **	26	53.1	21	35.0	47	43.1
Considering others more	9	18.4	4	6.7	13	11.9
“Talking it through”	9	18.4	6	10.0	15	13.8
Staying calm in conflict	12	24.5	15	25.0	27	24.8
Increased use of social support	4	8.2	3	5.0	7	6.4
**Improved self regulation**	11	22.4	10	16.7	21	19.3
Improved self confidence	5	10.2	2	3.3	7	6.4
Managing difficult emotions	3	6.1	6	10.0	9	8.3
**More helpful cognitions**	0	0.0	4	6.7	4	3.7
Beneficial but no specific example	0	0.0	7	11.7	7	6.4
Had skills prior to RAP-A	2	4.1	3	5.0	5	4.6
Haven’t needed skills	3	6.1	4	6.7	7	6.4
No/Not much/Don’t know	14	28.6	12	20.0	26	23.8

#### 3.1.2. “Talking it through”

A better developed ability to “talk it through” was reported by many students following RAP-A participation. Talking it through refers to the respondent’s ability to effectively and constructively work through a conflict with another individual. Participants gave many examples of being able to talk an issue through with their parents. For example: 

“*(I had) a bit of a disagreement (with Mum) and then I remember her (the RAP-A facilitator) telling me that I shouldn’t jump to conclusions and things like that and actually talk it through and that’s what I did. It turned out better than I expected*”.

Other examples include “*Now we (respondent and parents) just talk. I tell my sister just to talk about it and she’s like “okay”. So now she talks about it*”, and “*Everyone talks about how they deal with their parents. Like instead of fighting, just talk about it. If the music is too loud or whatever it is. Yeah we actually talked instead of yelling...yeah it was good*.” These responses also capture that being able to talk issues through with parents is a very positive experience for adolescents. 

Comments reflecting talking it through with peers were also common and the following examples illustrate the benefits of using assertive rather than passive or aggressive communication to talk an issue through: “*We had an assignment to do and one of the girls in my group hadn’t done her share and I asked her nicely instead of demanding and now she’s almost finished it*”, and “*I can remember in an argument with my friends... when we go to the RAP thing, you shouldn’t scream or anything, just calm yourself down a bit and talk through it...It’s not hard or anything and you can use it*”. 

This latter response demonstrates an overlap with the following sub-theme, staying calm in a conflict. It also highlights that belief that skills learnt during RAP-A were perceived as personally relevant and easy to use. 

#### 3.1.3. Staying Calm in a Conflict

Related to the ability to talk it through is the capacity to stay calm during a potential conflict. Many examples were given of the respondent managing to stay calm rather than behave aggressively when faced with conflict. An example of this that also captures some pertinent inner dialogue is, “*When we play football, if someone starts swearing, I think, “don’t worry, don’t hit him yet—work things out first”. Before I would have started punching way before*”. Other examples that illustrate how the individual was able to stay calm rather than lose their temper include: “*My Aunty and Uncle are living with us and we (respondent and her sister) have to share a room...she takes over my room and I didn’t yell at her...I just told her how I felt and said (she) had to clean up the room. Before I think I would have just blown it*”*.*

“*Me and a friend of mine, we had a bit of an argument about something...we didn’t argue about it, we just talked it through and got the whole thing fixed up. Before, we would have started yelling and thrown our tempers. I think it’s because of the RAP program. It gave me more confidence to do things I didn’t think I could do before*”. 

This final example again shows overlap with the previous sub-theme of talking it through.

#### 3.1.4. Increased Use of Social Support.

The theme of improved interpersonal relations also includes the important capacity to identify and utilize social support. Some respondents referred specifically to a RAP-A activity of identifying possible supports, for example, “*When I need someone to talk to, I just remember who I wrote down*”. Other responses made more general reference to the benefits of sharing difficulties with others in order to receive support, rather than keeping things to themselves, for example. “*Maybe just talking to people, not feeling so alone...(before) I would have bottled it up and kept it to myself*”. Seeking support to achieve specific goals was also reported by some participants, for example, “*At school, when I need help I go up to the teacher. I used to just sit there and try to do it myself. It’s been building up my marks because I know how to do it*”. 

### 3.2. Improved Self-regulation

The second major theme identified in the data was improved self-regulation; defining self-regulation as a process by which the self modifies its inner states and/or responses in a goal-directed manner [25]. Self-regulation usually involves overriding a response or behavior with a response that is more desirable [26]. A failure of self-regulation involves poor self and impulse control and has been implicated in many aspects of human functioning such as more psychopathology and greater levels of underachievement [27]. This theme of self-regulation is broad and as may be expected, has some overlap with improved interpersonal relations. Indeed, it is argued that improved self-regulation contributes to improved interpersonal relations, by assisting adolescents to keep calm during potential conflict and having a more well-developed capacity to communicate effectively and think clearly about difficult situations. However, improved interpersonal relations also seem likely to enhance self-regulation –though the development of more secure relationships and confidence in one’s ability to negotiate difficult interpersonal situations. The theme of self-regulation is divided into one sub-theme that contributes to improved self-regulation (improved self-esteem) and two specific examples of self-regulation (keeping calm and managing anger). 

#### 3.2.1. Improved Self-esteem

Responses appear to indicate that the strengths-based nature of RAP-A enhanced participants’ views of themselves, as illustrated by the comments, “*It just makes you feel like a better person*” and “*I used to think of myself, like, I’m nothing, but now instead of having those thoughts I think confidently about myself*”. This latter comment demonstrates a particularly powerful change for this individual. Another respondent elucidates how a specific RAP-A activity contributed to an improved self-esteem: “*I had really low self-esteem about myself earlier this year...but we did this group thing and we had to write down about other people, what they’re about and stuff and put it in an envelope and I still look at it and stuff. I kind of think what do other people think about me. That’s how my self-esteem boosts. When they write that about me—that I was nice, it really helped me out. I didn’t realize people thought of me that way*”. 

#### 3.2.2. Keeping Calm

An enhanced ability to manage stress and stay calm was noted by a significant proportion of respondents. Respondents reported that participating in RAP-A assisted them to find alternate ways of coping with problems and difficult emotions in order to stay calm, for example: “*Finding out more ways to cope with problems and calm down and stuff*”. Other examples of an improved ability to keep calm include: “*I was pretty stressed one night and I put on some really calm music and relaxed. Before I wouldn’t have done anything*” and “*That staying calm thing has helped me a lot... I used to go around hitting people if I got angry or bashing in the wall in my room and I used to just yell and yell. Now I listen to music*”.

These two comments capture the important ability to regulate one’s own emotions through engaging in a calming activity (e.g., listening to music). For the sake of clarity, we note that responses specific to interpersonal situations are not included in this sub-theme and are instead captured in the sub-theme, “staying calm in a conflict”. 

#### 3.2.3. Managing Anger

Better management of anger was another common response, particularly amongst male respondents. Although this theme overlaps somewhat with “keeping calm”, these themes were separated out to recognize their high frequency. A number of participants reported having developed new ways of responding to anger and/or aggressive impulses, for example, “*...like when you’re angry and all that, think about how to calm yourself down*”, “*It teaches you to sort it out in different ways which is better than just going off...*”, and “*You just take the time to think. Like if you’re going to get angry you think, “What’s the consequences?”*”. These responses illustrate an improved ability to exert control over the expression of anger, and in doing so, deal with this difficult emotion in a healthier manner.

### 3.3. More Helpful Cognitions

Although considerably less prevalent than the previous two themes, a third theme was identified in the data—more helpful cognitions. A number of participants gave responses indicating that they have developed the ability to “find the positive” in potentially difficult situations. For example, “*Yeah I think of the positive side of things. I’ve got into trouble or something, I just think of the positive*”. Alongside this is the perspective that seeing the positive is beneficial to the individual and is something the participants are trying to do e.g., “*I try to think more positive now...*”. One respondent explains the change they have noticed about themselves: “*I used to be pretty pessimistic about stuff, used to think everything bad is going to happen but now I’ve changed it a bit*”. Another respondent gives a pertinent example of trying to identify more helpful cognitions about a situation: “*Like not thinking negatively, like if someone stands you up, don’t think they don’t like you, just think they missed the bus or something. Don’t blame them. I was with my girlfriend and she stood me up twice and I just thought it doesn’t matter because she says she likes me and I trust her and I shouldn’t think she doesn’t like me or something and I shouldn’t think negatively*”. 

## 4. Discussion and Conclusions

This study used qualitative methodology to explore a range of perceived benefits of adolescents’ participation in RAP-A, an evidence-based depression prevention program. Over one-half (54%) of interviewed adolescents could articulate specific examples of program benefit in their daily lives. The two major themes that arose in analysis of the data were improved interpersonal relationships and improved self-regulation, in addition to one minor theme—more helpful cognitions. Nested within the theme of improved interpersonal relationships were the sub-themes improved empathy, “talking it through”, staying calm during a conflict, and increased use of social support. Sub-themes included within improved self-regulation were improved self-esteem, keeping calm, and managing anger. 

It seems important to reflect upon the potential relations between the themes and sub-themes. During analysis a degree of overlap was noted amongst a number of the sub-themes and across the broader themes. This was deemed unsurprising considering a strong reciprocal relationship appears to exist between the two major themes. Indeed, the evidence to date suggests that interpersonal relationships impact upon an individual’s ability to successfully self-regulate [26,27], and the consequences of effective self-regulation are likely to extend to enhanced interpersonal relationships [28]. More helpful cognitions are also purported to assist in effective self-regulation [29]. 

The two major themes that were identified are particularly interesting in light of assumed mechanisms of change in such universal programs. Many programs include CBT components, as does RAP-A. However, RAP-A also includes components drawn from interpersonal approaches and interestingly, one of the major themes uncovered in this study echoes this. Although there was a minor theme reflecting cognitive changes, a much greater proportion of responses were about relationships with others and an ability to better manage these. This was somewhat surprising given the program included a strong CBT component. Interpersonal difficulties play a significant role in depressive disorders e.g., [30,31] and there is increasing research to suggest interpersonal factors may be particularly germane to depression e.g., [32]. Although the cognitive dimensions were less frequently identified, their influence may well be expressed through improved self-regulation and interpersonal skills. This hypothesis is supported by a randomized trial that compared two depression prevention programs—one cognitive-behavioral and the other based on interpersonal psychotherapy—and a control condition [12]. This study found non-significant mediation effects on the cognitive variable of attribution style.

Future research of universal prevention programs may benefit from exploring changes in self-regulation and improved relationships with others, in addition to typically evaluated outcomes such as depressive symptoms. In broadening the range of outcomes we assess, it is necessary to consider the measures that may best measure the impact of these interventions. It may prove difficult to find appropriate psychometric measures to tap into the range of program benefits as currently used measured may be unsuitable or assess too narrow a range of outcomes. Broadening our measurement of program outcomes will also allow exploration of potential mechanisms of change. Further research exploring potential mediators, such as the themes identified in this study, are likely to advance our understanding of these important interventions. 

Several limitations are present in the present study. The selection of participants to this qualitative part of the study was unlikely to be truly random as it was carried out by year level coordinators at each school, and is likely to have been influenced by matters of convenience. With regard to the conduct of the interviews, a lack of asking probing questions following responses that were short, unclear, or lacking in detail may have contributed to the considerable percentage of participants (46%) who were unable to articulate a specific benefit of the program. For example, students who indicated that RAP-A was beneficial but did not refer to a particular benefit may have been able to give more detailed responses with further probing. Another limitation is the lack of data triangulation; it would also have been valuable to obtain convergent information from significant others such as parents, teachers and peers. It is also important to consider whether participants’ responses were influenced by a desire to please the interviewer with “correct” answers. Indeed, it is plausible that the extracted themes reflect the content of RAP-A, which might be expected given the underpinnings of the program. However, the interviewer was unknown to the participants and reassurance was given that both positive and negative feedback would be useful. 

In conclusion, students appear to acquire a range of skills from universal depression prevention programs and make use of them in different situations. As such, we do a disservice to adolescents if we restrict our interest in program outcomes to a reduction in depressive symptoms. In the current study improved interpersonal relationships and improved self-regulation appear to encompass the most significant perceived program benefits in adolescents’ daily lives, and it is suggested that these variables have a reciprocal relationship. It seems important to continue exploring such potential mechanisms of change of depression prevention programs in order to inform best practice interventions.
